# Assessing spirituality: is there a beneficial role in the management of COPD?

**DOI:** 10.1038/s41533-019-0134-x

**Published:** 2019-05-28

**Authors:** Irini Gergianaki, Maria Kampouraki, Siân Williams, Ioanna Tsiligianni

**Affiliations:** 1 Health Planning Unit, Department of Social Medicine, University of Crete, School of Medicine, Heraklion, Greece; 2 Moires Health Care Center, Crete, Greece; 3 International Primary Care Respiratory Group, London, UK

**Keywords:** Health care, Health services

## Abstract

Here,we report on the role of spirituality assessment in the management of chronic obstructive pulmonary disease (COPD). Although a positive effect of addressing spirituality in health care has been proved in a number of chronic diseases, its potential in COPD has received less attention. Although limited, available evidence suggests that spirituality may play an important role in improving quality of life of patients with COPD. The fruitful results in other long-term conditions may lay the foundation for further research on addressing spirituality in COPD. This should focus where the burden of COPD is greatest, including low-resource settings globally. Implementation research should include exploration of an acceptable consultation process to identify patients who would welcome spiritual discussions; how to integrate spiritual approaches into health care professional curricula so that they are aware of its importance and have the confidence to raise it with patients and how to integrate spiritual approaches into holistic COPD care.

## Introduction

Chronic obstructive pulmonary disease (COPD) is a complex, heterogeneous disease characterized by persistent airflow limitation and repeated exacerbations.^1^ The burden of COPD at the individual and societal level is growing and despite progress in survival rates, the disease is associated with substantial life quality impairment.^2^ Distressing symptoms, dependence on others, emotional problems and social restriction are among the reasons why many patients with COPD face an overwhelming difficulty in coping with the disease.^3^ Particularly in more severe stages of COPD, death’s proximity can be a serious struggle for patients and their families.^4^ Such challenges represent an important unmet need in disease management, especially in primary care settings.

The positive effect of addressing spirituality in health care has been explored and proved in a number of chronic diseases such as cancer, kidney disease, mental disorders, and AIDS.^5–9^ Yet, during the last decade, the potential role of spirituality in chronic lung diseases such as COPD has received more attention, with a number of studies suggesting that spiritual approaches may be helpful in improving the physical, mental, and social status of patients.^10,11^ Unfortunately, health care professionals (HCPs) often are not aware of the importance of spirituality and, either due to lack of time or adequate skills, do not properly address and include it in their clinical routine.^12^ Consequently, the field of spirituality remains rather neglected in COPD, research is limited and, most importantly, training, either in various health disciplines’ curricula or in continuing education, is inadequate.^13,14^

The present perspective article aims to summarize the current evidence regarding the context and role of spirituality in managing patients diagnosed with COPD, as well as the ways HCPs could reflect upon the spiritual needs of these patients. It is set in the context of the burden of COPD, where the vast majority of those dying are in low- and middle-income countries^15^ where health care assets are most limited, and therefore where the role of religion and spiritual beliefs may have a significant place.^16^

## Spirituality: an old term with a new concept

The word “spiritual” derives from the Latin world “*spiritualis*” which is a translation of the Greek word “*pneumatikos*” (pneuma = spirit), as it appears in Paul’s letters to the Romans and Corinthians.^17^ It is noted that healing has been associated with spirituality since the days of Hippocrates.^18^ Today, spirituality in health care is still relevant, having of course expanded beyond its original meaning to include any belief system focusing on intangible elements that bring meaning to life.^19^ Interestingly, spirituality has been consistently found to have significant impact on the occurrence and outcomes of many chronic diseases,^5–9^ affecting different aspects of the physical and psychological life of patients and clinicians, improving their self-confidence, communication, problem-solving, and decision-making.^20,21^

### How is spirituality defined?

Spirituality can be defined as an aspect of humanity referring to the way people search for and express purpose and meaning and, more specifically, “the way they experience their connectedness to the moment, to self, to others, to nature, and to the significant or sacred.”^20^ Spirituality and religion are commonly regarded as similar or even the same,^18^ although the two concepts differ substantially. Religion can be considered as a more organized/formalized way in which people express their spirituality. Therefore, it represents a subcategory of spiritual experience.^18^ On the other hand, spirituality is not linked to a particular doctrine and, in health care terms, it mainly constitutes an adaptive/coping mechanism.^22^ In particular, spiritual coping refers to a constant adaptation of an individual’s efforts, either on cognitive or behavioral grounds, to manage stressful events or conditions.^23^ Thus, patients with strengthened spirituality are able to use various coping and adaptive strategies such as plan-based problem-solving, distancing, positive reappraisal, self-control and accepting social support, among many others.^23^

### The impact of spiritual approaches in chronic diseases

The research interest in the impact that spiritual approaches may have on the outcomes of chronic diseases, has been revitalized during the last decade.^9,24^ A recent meta-analysis of randomized controlled trials in patients with cancer, reported that spiritual interventions improved quality of life and decreased depression and anxiety, as well as the feeling of hopelessness.^25^ Similarly, a systematic review reported that higher levels of spirituality may be related to better quality of life among patients with cardiovascular diseases^26^. There is also evidence suggesting positive association between spirituality and health outcomes of patients with end stage renal disease.^27^ Further, a robust belief system was found to be positively associated with coping skills in patients with schizophrenia,^23^ among other mental illnesses.^9^ Lastly, Kremer et al. suggested potential psychoneuro-immunological mechanisms, as they observed that spiritual coping among patients with AIDS leads to slower HIV disease progression (CD4 cells preservation and sustained undetectable viral load, defined as the number of HIV RNA copies per milliliter of blood), as well as more positive health behaviors, such as adherence to therapy and safer sexual behavior.^8^ Collectively, the evidence presented supports the high value of considering the impact of spirituality in the management of chronic diseases.^28^

## Why do we need a spirituality-based approach in COPD?

Interestingly, most patients have spiritual as well as physical health concerns, so spiritual health should not be neglected in clinical routines. ^9,29^ Although spiritual needs may increase during the chronic illness course, spiritual care for patients with COPD has been scarcely discussed in the literature.^30^ Probably, because the mechanistic/biological view of the human body and disease is the dominant perspective in current Western medical practice, and therefore has been most researched, and despite the revised interest in patient-centeredness, the religiosity/spirituality-based approach has lagged behind.^21^

At this point we have to note that due to globalization, the dichotomy between Eastern and Western medicine has more blurred boundaries, yet there is still a substantial difference in the value that each gives to spirituality.^31^ Thus, traditional Eastern medicine is mainly based on a philosophy of a holistic view of the body, which is conceptualized as an integral part of the social-natural environment.^31^ This concept is embedded in a general cultural approach of keeping a balanced life status and being mindful.^31^ This has advantages in COPD management, with promising results from mindfulness-based interventions added in pulmonary rehabilitation (PR).^32^

Importantly, literature reveals the desire of patients for their religion/spiritual needs to be included in their medical care, especially at the advanced stages of their disease.^33,34^ Properly incorporating spirituality into every day clinical routine can have significant positive effects on patients by providing comfort, increase adherence to treatment, and improve the quality of life.^35^ In addition, reducing patients’ fears and anxiety may in turn lead to less health care utilization and fewer hospitalizations.^36^

## What are the benefits of spiritual-based approaches in COPD?

COPD is a disease with a chronic, disabling course that strongly impairs functional and mental status.^1^ Hasegawa et al.^30^ showed that people with advanced COPD stage III or IV experience a spiritual state which is similar to that of people with inoperable lung cancer. In particular, there were no significant differences between these two groups of patients either in median FACIT-Sp-12 (Functional Assessment of Chronic Illness Therapy-Spiritual Well-Being scale) scores or in subscales estimating meaning/peace and faith.^30^ Υet people with COPD appeared to have a worse support state than those with lung cancer, highlighting the need to raise awareness of spiritual care in advanced COPD.^30^

Spirituality possibly relates to better outcomes, following other COPD management strategies as was shown in a study by da Silva et al.^37^, which presented the results of a nonrandomized controlled clinical trial that examined religious coping (RC) (defined as the “use of behavioral/cognitive techniques in stressful life events”) and religiosity strategies (“use of individual beliefs, values, practices, and rituals related to faith”) of people diagnosed with COPD who attended a PR program.^37^ RC was assessed with the “Brief Religious Coping questionnaire (Brief-RCOPE), a 14-item scale distinguishing between positive RC (PRC) and negative RC (NRC)—religious struggle—styles. More specifically, the positive changes in PRC that were observed after PR were weakly but statistically significantly correlated with improvement in the 6-min walking test (6-MWT),^37^ a measurement of exercise capacity, and inversely associated with COPD Assessment Test (CAT test), which analyses the impact of disease symptoms.^37^ Additionally, improvements in NRC after PR were inversely and moderately correlated with changes in patient health questionnaire-9, which assesses severity of depressive symptoms.^37^ Lastly, organizational religious activity—which is relevant to the frequency of attendance at religious services—was weakly positively associated with improvements in 6MWT and weakly negatively correlated with CAT.^37^ A limitation of this study was that a convenient (not randomized) sample was used.^37^ In addition, it should be noted that the mechanisms of these associations are unclear and possibly not isolated to religiosity.

In a third study, spirituality measured with the spiritual transcendence scale was strongly associated with better quality of life (Quality of Life Index–Pulmonary Version III, QLI-PV) and weakly but significantly associated with lower stress in patients with COPD.^38^ Importantly, we have to note that no association was reported between spirituality and feeling of breathlessness, impairment at functional level, or pulmonary function tests.^38^ In the same study, when spirituality was conceptualized as a sense of coherence, measured with the short form of orientation to life questionnaire, it was strongly correlated with improved quality of life, lower stress, and decreased shortness of breath as reported by patients with COPD, yet it was not related to objective measures.^38^ This was a cross-sectional study through mailed questionnaires (*n* = 182 patients with COPD, mostly Caucasians, Christians, and well educated, with 8 years mean duration of the disease), with a number of limitations, such as low response rate, and possible self-selection of the participants.^38^

Collectively, the findings described above highlight important opportunities for continued exploration of the relationship between spirituality and quality of life in individuals with COPD. This is particularly relevant given the inclusion of assessing quality of life in the Global Initiative for Chronic Obstructive Lung Disease Guidelines and its potential clinical impact on the classification of patients into lower-COPD risk categories.

## Explaining the assosiation of spirituality with health

Like other factors promoting health such as physical exercise, religiosity/spirituality possibly has positive effects on disease outcomes through the interaction of multiple mediators. Characteristically, religiously involved individuals are more likely to adopt health-promoting habits, such as healthy eating or fasting and avoid high risk behaviors, such as smoking, which, consequently, may have a positive effects on COPD prognosis.^39^ Second, religious and spiritual practices (such as meditation and prayer) create positive emotions, such as hope and forgiveness, which may be important for people with COPD who tend to self-blame regarding their disease.^40^ Positive emotions may also ameliorate anxiety/depression and even have physiological effects (i.e., decrease blood pressure/heart rate and oxygen consumption). Lastly, a fascinating field is emerging, investigating neural mechanisms associated with spirituality, which could possibly delineate a more exact biological mechanism in the future.^41^

## Do physicians assess spirituality in patients with COPD?

Although, a number of guidelines^42,43^ and organizations suggest a spiritual assessment of patients, practising HCPs do not usually implement such an approach in their routine practice.^44^ There is a fine line for a HCP to cross in discussing religion or other spiritual experiences with their patients. A recent study confirms that many HCPs do not discuss spiritual issues and, when they do, patients with COPD rate the quality of discussions relatively poor.^45^ On the other hand, neglecting patients’ beliefs may lead to deterioration of their care, as spirituality may constitute a vital part of their coping ability, and hence, their overall wellbeing.^46^ However, if any spiritually based approach is not performed ethically, patients may complain.^47^ It is notable that HCPs who were older, female, nonwhite, more religious among HCPs interviewed, with more professional experience, or in family medicine have more positive attitudes toward taking a spiritual history and consequently, are more likely to perform this assessment.^48^ In addition, religious HCPs who have more deeply integrated their beliefs into their medical practice, are most likely to understand how religion is relevant to medical care and take appropriate action.^21,29^

## For which patients with COPD is spiritual care valuable?

As the severity of COPD increases, the proportion of patients welcoming an inquiry about their spiritual beliefs increases from 33% in an outpatient office visit, to 40% if hospitalized, and up to 70% at the end of life.^49^ Settings with fewer time pressures, which may also provide an opportunity to involve family members in the discussions, are more amenable to a spiritual-based approach.^49^

Ehman et al.^33^ found that 45% of patients in an outpatient pulmonary clinic held spiritual/religious beliefs that would influence medical decisions at a more advanced stage of their disease, especially at palliative care or end of life care. The need to support patients with COPD is greater in those with advanced spirometric stages (stage III or IV), who experience lower levels of spiritual well-being.^30^ As expected, at these stages, patients with COPD present with more anxiety, fears, and depression.^30^ Inevitably, for the person with very advanced COPD or dying, the need for sharing their spiritual worries becomes extremely significant.^50^ Thus, it would be helpful, if treating HCPs gradually increased the amount of spiritual discussion with patients diagnosed with COPD and, if appropriate, also with their families. It is promising that where hospices already admit patients with COPD, they provide spiritual support but more research is needed to advance the field.^50^

Also, it should be noted that patients’ preferences for end-of-life conversation vary. In one study, 45% of patients with COPD reported that religious beliefs would influence their health-related decisions if gravely ill.^33^ In the same study,^33^ 94% of the responders with spiritual beliefs vs. 45% of patients without such beliefs, accepted that HCPs should ask about their spiritual needs. Overall, two thirds of the patients indicated that they would welcome a spiritual question in their medical history.^33^ Daaleman et al. in a qualitative study also found that the frequency of patients’ participation in religious activities was related to their acceptance of HCPs’ inquiry regarding their religion/faith.^51^ Therefore, health care professionals should respect those not willing to be involved in a spiritual conversation and tailor discussions to those who accept.^52^

## Are HCPs educated about spirituality?

The HCP-patient interaction is unique, compared with other social or professional interactions, as it addresses personal or sensitive information. There is a variation in physician beliefs about the relevance of religion/spirituality for patient recovery.^53^ In a survey of 2000 practising HCPs, only 1% of them considered that they spend too much time on religiosity/spirituality conversations, 38% that they spend too little time and 61% “the right” amount of time.^54^ More specifically, reasons reported by physicians for not addressing spiritual issues include lack of time or training, and difficulty in identifying the patients who would welcome spiritual discussions.^55^. Nurses also report lack of time, confidence, or comfort to explore these issues with patients.^56^ According to a relevant qualitative study^57^ members of palliative care teams who are more involved with spiritually related activities reported that they helped not only the patients, but also palliative care team members to form bonds with each other, patients and patients’ families and search for meaning and hope in illness and suffering.^57^

## How to advance spirituality based approaches to manage patients with COPD?

The fundamental requirement for inclusion of religiosity and spirituality in clinical routines is a patient-centered approach.^58^ Koenig et al.^59^ reported that although 90% of U.S. medical schools have individual lessons on spirituality related to health and disease, only 7% offer dedicated courses. The spiritual involvement of HCPs should be added early on in their curricula, and continued throughout their training. Moreover, guidelines on COPD could share the information about spiritual care and good practice from other chronic diseases, where spirituality has been successfully used, to help patients improve their health status and well-being and recommend further research in COPD so that firm recommendations can be made.^42,43^ In the meantime, taking in consideration the positive associations that spirituality has on health status and quality of life (and potentially patients’ disease classification), it seems reasonable to suggest to further explore the usefulness of integrating a spiritual based approach into guideline recommendations.

## Summary

While few studies have investigated the role of spirituality in the management of patients with COPD, available evidence suggests that spirituality may play an important, yet neglected, role in improving quality of life for these individuals. The fruitful results regarding the impact of spirituality in populations with other chronic diseases may lay the foundation for further research focused on COPD. This needs to focus where the burden of COPD is greatest, which includes low-resource settings globally. Improving HCPs training, confidence, and ability to identify patients who would welcome spiritual discussions are the possible necessary first steps toward their engagement in a more spiritual and, consequently, more holistic approach to COPD management.
